# Selection for Genetic Variation Inducing Pro-Inflammatory Responses under Adverse Environmental Conditions in a Ghanaian Population

**DOI:** 10.1371/journal.pone.0007795

**Published:** 2009-11-11

**Authors:** Maris Kuningas, Linda May, Riin Tamm, David van Bodegom, Anita H. J. van den Biggelaar, Johannes J. Meij, Marijke Frölich, Juventus B. Ziem, Helena E. D. Suchiman, Andres Metspalu, P. Eline Slagboom, Rudi G. J. Westendorp

**Affiliations:** 1 Department of Gerontology and Geriatrics, Leiden University Medical Center, Leiden, The Netherlands; 2 Department of Biotechnology, Institute of Molecular and Cell Biology, University of Tartu, Tartu, Estonia; 3 Telethon Institute for Child Health Research, Center for Child Health Research, University of Western Australia, Perth, Australia; 4 Leyden Academy on Vitality and Ageing, Leiden, The Netherlands; 5 Department of Clinical Chemistry, Leiden University Medical Center, Leiden, The Netherlands; 6 School of Medicine and Health Sciences, University of Development Studies, Tamale, Ghana; 7 Department of Molecular Epidemiology, Leiden University Medical Center, Leiden, The Netherlands; 8 Estonian Genome Project of University of Tartu, Tartu, Estonia; 9 Netherlands Consortium for Healthy Ageing, Leiden, The Netherlands; Ohio State University Medical Center, United States of America

## Abstract

**Background:**

Chronic inflammation is involved in the pathogenesis of chronic age-associated, degenerative diseases. Pro-inflammatory host responses that are deleterious later in life may originate from evolutionary selection for genetic variation mediating resistance to infectious diseases under adverse environmental conditions.

**Methodology/Principal Findings:**

In the Upper-East region of Ghana where infection has remained the leading cause of death, we studied the effect on survival of genetic variations at the *IL10* gene locus that have been associated with chronic diseases. Here we show that an *IL10* haplotype that associated with a pro-inflammatory innate immune response, characterised by low IL-10 (p = 0.028) and high TNF-α levels (p = 1.39×10^−3^), was enriched among Ghanaian elders (p = 2.46×10^−6^). Furthermore, in an environment where the source of drinking water (wells/rivers vs. boreholes) influences mortality risks (HR 1.28, 95% CI [1.09–1.50]), we observed that carriers of the pro-inflammatory haplotype have a survival advantage when drinking from wells/rivers but a disadvantage when drinking from boreholes (p_interaction_ = 0.013). Resequencing the *IL10* gene region did not uncover any additional common variants in the pro-inflammatory haplotype to those SNPs that were initially genotyped.

**Conclusions/Significance:**

Altogether, these data lend strong arguments for the selection of pro-inflammatory host responses to overcome fatal infection and promote survival in adverse environments.

## Introduction

Up to a few generations ago, human life histories were shaped under harsh environmental conditions with high pathogenic burden that led to selection for individuals who could resist fatal infection [1]–[4]. We consider it likely that in contemporary populations living under adverse environmental conditions such responses are still essential for survival. Selection for a genetic make-up promoting resistance against infections may be observed at cytokine genes. Previously, genetic variants at the *IL10* locus have been shown to modulate innate inflammatory responses, and associated to chronic diseases [5]–[11]. However, the proposed evolutionary selection for variants mediating resistance to infectious diseases has been taken for granted [12]–[14]. We investigated such selection in action in group of people from a large contemporary rural population (n = 25,184) living in a remote Garu-Tempane district, a densely populated agricultural area in southeast of the Upper-East region of Ghana. This population lives under adverse environmental conditions characterized by high infectious pressure and high mortality rate, especially at young age [15].

## Results and Discussion

**Figure 1:**
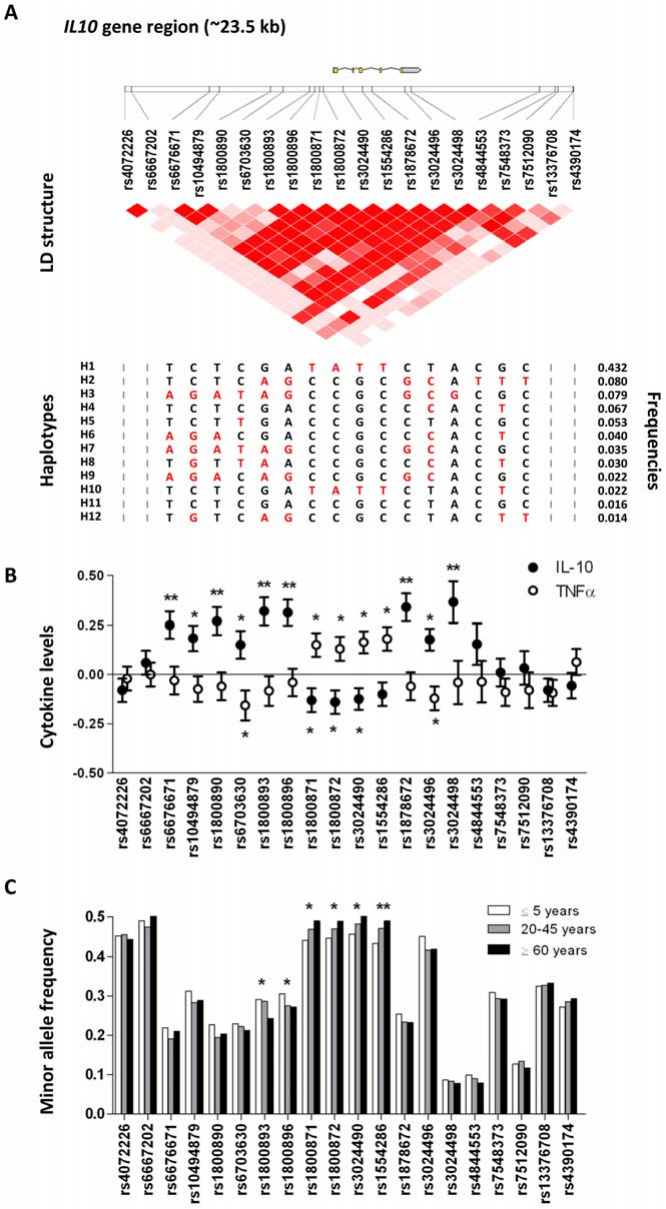
*IL10* gene structure, cytokine production and changes in allele frequencies with age. (**A**) *IL10* gene structure with flanking gene regions (∼23.5 kb) and location of the genotyped SNPs. Pair-wise linkage disequilibrium (LD, D') as observed in the Ghana study population (n = 4336) is also depicted. Frequencies of the different haplotypes (if >1%) are presented with the minor alleles of each SNP indicated in red; (**B**) The association between the minor allele of each *IL10* SNPs and *ex vivo* cytokine production in response to co-stimulation with LPS and zymosan (n = 615). The production of IL-10 and TNFα is expressed as z-scores with s.e.m, which indicate the deviance from population mean (zero-value) for carriers of at least one copy of the minor allele. Data were obtained using linear regression adjusted for age, sex, socioeconomic status and tribe, * p<0.05; ** p<0.001; (**C**) Minor allele frequencies of the *IL10* SNPs in three age groups: ≤5 years (n = 1014), 20-45 years (n = 1462) and in ≥60 years (n = 727). Differences between groups were studied using linear regression adjusted for sex, socioeconomic status and tribe; * p<0.05; ** p<0.001.

IL-10 is a potent immunoregulatory cytokine that inhibits the synthesis of various pro-inflammatory cytokines [16], [17]. Several SNPs in the *IL10* gene are known to influence innate cytokine production. Here, we genotyped these and additional SNPs in the *IL10* gene, in total 20 SNPs, covering all genetic variation in the coding, upstream and downstream regions (∼23.5 kb) (**Figure 1A** **;** **table S1**) in 4336 participants, and tested their influence on cytokine production and survival in different environmental conditions. The allelic frequencies were in Hardy-Weinberg equilibrium for most of the SNPs, with few exceptions where minor deviations were observed (**table S1**). The majority of genotyped SNPs fell into one linkage disequilibrium (LD) block, which is characterized by one common haplotype (haplotype 1, population frequency 43%), and by several minor haplotypes (frequencies≤8%, **Figure 1A**). IL-10 and TNF-α production (n = 615) were found to be influenced by a number of SNPs. Haplotype 1 that constitutes of the rs1800871, rs1800872, rs3024490 and rs1554286 SNPs was associated with lower IL-10 (p = 0.028) and higher TNF-α (p = 1.39×10^−3^) responses than the population mean, i.e. with a pro-inflammatory response (**Figure 1B** **;** **tables S2** **,** **S6**). The low TNF-α response that is found in parallel with high IL-10 response reflects the IL-10 mediated down regulation of TNF-α. This observation is in accordance with previous studies showing that increased levels of IL-10 limit inflammatory response to minimum, and keep collateral damage at bay [17]. In case of haplotype 4 and 5, which also led to lower IL-10 production, but not to lower TNF-α production, it might be reasoned that the contribution of these *IL10* variants to low IL-10 levels are not very strong, as reflected by large error margins. This might also explain why the TNF-α levels are low, as the opposite would have been expected.

Next, we compared allele frequencies of the various *IL10* SNPs and haplotypes between children (≤5 years), middle aged (20–45 years) and older (≥60 years) individuals to determine whether some of these alleles are enriched or depleted with age. The discriminating SNPs and haplotype 1 that associated with pro-inflammatory cytokine response pattern, were significantly enriched among older Ghanaians (p_trend_ = 2.46×10^−6^ for haplotype 1), hence reflecting a survival benefit for carriers of these alleles up to relatively high ages (**Figure 1C** **;** **tables S3** **,** **S7**).

**Figure 2:**
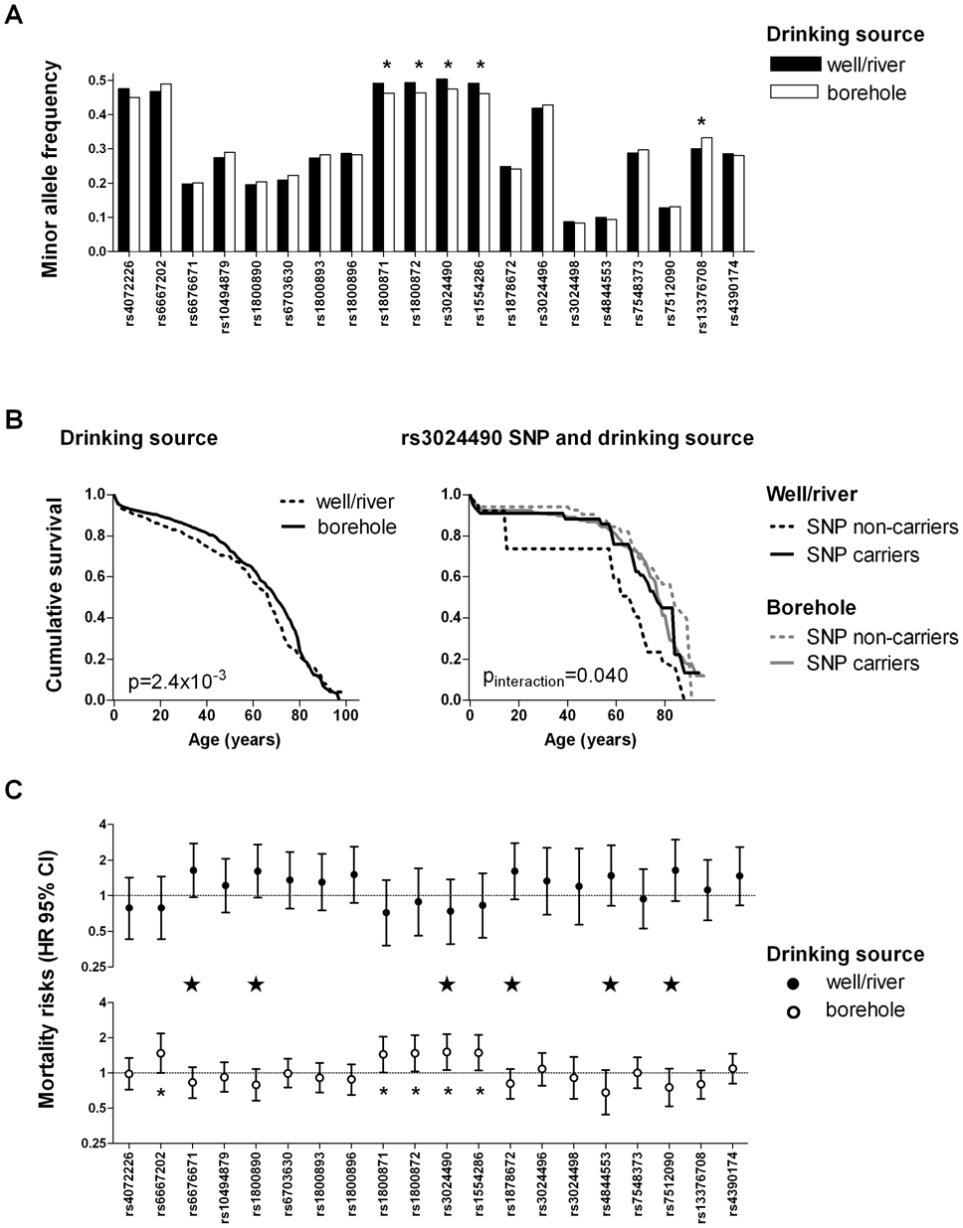
Environmental interaction with gene expression in Ghana. (**A**) Minor allele frequencies of the *IL10* SNPs for people who make use of wells/rivers (n = 802) or boreholes (n = 3284) as a drinking source (* p<0.05); (**B**) Age-specific mortality risks during a five-year follow-up period for people drinking from wells/rivers and in interaction with the rs3024490 SNP. People drinking from wells/rivers had higher age-specific mortality risk compared to those drinking from boreholes (hazard ratio (HR) 0.79, 95% CI [0.67–0.92], p = 3.0×10^−3^). In addition, carriers of the pro-inflammatory rs3024490 SNP experienced a survival benefit when using water from wells/rivers but suffered a slight mortality risk when boreholes were used as a drinking source (p_interaction_ = 0.040); (**C**) Mortality risks for carriers of the *IL10* SNPs separately for those who make use of wells/rivers, or boreholes, as a drinking source and interaction terms (indicated with a big star, p<0.05).

From 1973 to 1980 the Ghana Water Utilization Project drilled boreholes throughout Ghana [18], which resulted in a major quality improvement of drinking water over the last 25 years. In the research area (n = 25,184) the majority of people now have access to safe drinking water from boreholes (80.6%), whereas a minority still retrieves their water from traditional drinking sources, such as wells (17.8%) and rivers (1.5%). The adverse effect of unsafe drinking water is reflected by a significantly higher mortality risk among people using rivers/wells as a drinking source compared to those having access to boreholes (hazard ratio (HR) 1.28, 95% CI [1.09–1.50], p = 2.4×10^−3^, **Figure 2B**). We tested whether the source of drinking water provides a selection pressure for pro-inflammatory genetic variation at the *IL10* locus (**Figure 2A**; **table S4**). Indeed, the frequency of haplotype 1 (p = 8.20×10^−3^) and the minor alleles of each of the relevant SNPs (rs1800871, rs1800872, rs3024490 and rs1554286, p<0.05) were significantly higher among people using drinking water from wells/rivers compared to those who use water from boreholes (**tables S4** **,** **S8**). Furthermore, during five year follow-up period, we observed a significant interaction between drinking source and genetic variants in the *IL10* gene on survival (**Figures 2B, 2C**). Carriers of pro-inflammatory SNPs and haplotype 1 experienced a survival benefit when using water from wells/rivers but suffered a mortality risk when boreholes were used as a drinking source (for haplotype 1 p_interaction_ = 0.013) (**tables S5** **,** **S9**). Similar association were observed when using data for people who have drunk for their entire lives from boreholes (n = 1296) or from wells/rivers (n = 347)(**tables S10** **and** **S11**). This data demonstrates that pro-inflammatory alleles rescue their carriers of excess mortality in adverse environmental conditions leading to survival probabilities that are comparable to those in affluent environments, and thereby creating selection pressure. However, in an affluent environment, the same pro-inflammatory response leads to increased mortality.

Inflammation is already known to be a double edged sword as there is always collateral damage when fighting infection. In case of malaria, it has been observed that an intermediate response provides a defence against infection whereas an excessive response increases the likelihood of a fatal outcome [19]–[21]. In addition, it is likely that a subtle balance in the selective pressure for pro- and anti-inflammatory alleles exists to outweigh positive over negative effects. Under more benign conditions, the adverse effects may become more prominent, as altered pathogen loads, e.g. due to improved drinking water, could disturb the fine-tuning between immune response and environment. Studies have demonstrated that chronic infection with helminths of which many are water-borne, have a downregulatory effect on immune responsiveness, both innate [22] and adaptive [23], whereas removal of helminths and their anti-inflammatory influence lead to pathological conditions [24]. In a population, where infections are prevalent (in the Ghanaian population 85% of all participants and 100% under 30 years of age are infected with *P. falciparum*), decrease in downregulatory capacity might have a detrimental impact on the course and outcome of infection as a result of excessive inflammatory reaction [24].

**Figure 3:**
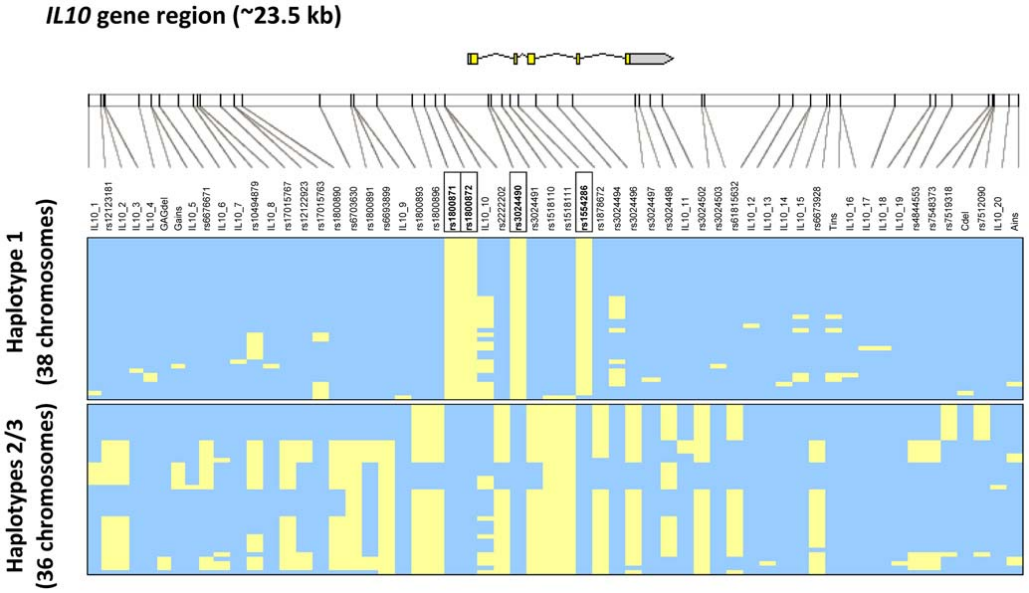
Location of genetic variants identified by resequencing the *IL10* gene, and haplotype structure as inferred by PHASE. Haplotypes were obtained from unphased resequencing data for 74 chromosomes (37 individuals) and were grouped into haplotype 1 and haplotypes 2 or 3 based on the initial criteria for selecting individuals for resequencing. Characteristic of the originally selected haplotype 1 was the presence of the rs1800871, rs1800872, rs3024490 and rs1554286 SNPs (indicated in boxes). The major and minor alleles are indicated in blue and yellow, respectively.

Next we set out to investigate whether the pro-inflammatory (low IL-10/high TNF-α) response could originate from variants in the *IL10* gene other than the rs1800871, rs1800872, rs3024490, and rs1554286 SNPs within the haplotype 1. It could be that these variants tag some nearby functional variant. Hereto we resequenced the *IL10* gene region (∼23.5 kb) in 37 individuals, of whom 19 carried haplotype 1, and 18 haplotypes 2 or 3 (**Figure 3**). The resequenced region extended beyond LD boundaries as observed in the HapMap Yoruba data. In total, we identified 58 variants of which 33 were previously identified SNPs (present in dbSNP) and 20 undescribed SNPs. In addition, we observed three insertions and two deletions (**table S12**). In contrast to expectation, haplotype 1, which has a population frequency of 43%, did not contain any additional common variants to the four SNPs that were initially genotyped (**Figure 3**). These suggests that one or more of these SNPs are likely to be functional themselves or are in LD with a functional variant that leads to lower IL-10 production as observed earlier. The latter has been commonly suggested by studies analyzing *IL10* gene variants. However, since we resequenced an *IL10* gene region beyond LD boundaries, then this option is unlikely. It is far more likely that these variants influence *IL10* transcription themselves. Previous experimental data has shown that rs1800871 SNP resides in a binding site for GATA3, which directly remodels the *IL10* locus, and that rs1800872 SNP resides in the binding site for ETS1, influencing transcription activity of *IL10* [25]–[27].

Variation in the *IL10* gene has been extensively studied in relation to cytokine production and disease prevalence. In line with our reasoning, several *IL10* variants have been associated with increased prevalence of inflammation-related diseases, but other studies have reported no or opposing associations [5]–[7], [10], [11], [28], [29]. One possible explanation is that regulation of the *IL10* gene may be different in cells of the innate and adaptive immune system, and genetic associations may consequently differ for various pathologies. In addition, population differences might be at play as the allele frequencies of *IL10* SNPs differ between populations: for instance, the minor alleles of rs1800871 and rs1800872 occur at lower frequencies in Caucasian than in African populations (rs1800871: 17.3% vs. 47.0%; rs1800872: 20.2% vs. 47.2%, **Table S1**). This could reflect the populations' demographic history, or be the result of relaxed selection on resistance to infection, as in affluent environmental conditions these alleles are not essential for survival anymore. That could have lead to accumulation of additional genetic variants in the *IL10* gene locus in Caucasian populations, thereby providing an alternative explanation for the opposing results obtained by various studies.

In summary, the association between genetic variation in the *IL10* gene, cytokine production and mortality, the skewing of allele frequencies over age and environmental conditions, all provide strong arguments for selection of genetic variation inducing pro-inflammatory responses. Population stratification is unlikely to explain for the associations observed, since analysis of mtDNA and Y-chromosomal genetic variation patterns in our study population revealed that female mediated gene flow is nearly fully random whereas male mediated gene flow is highly reduced. This genetic substructure is an immediate result of the patrilocal society of the study population. In addition, in all our analyses we controlled for socioeconomic status and tribe, variables that may have influenced the associations found. Altogether, our data support the hypothesis that selection for individuals with a potent innate, pro-inflammatory host response is favoured under adverse environmental conditions to fight infection. However, the same response has increasingly been recognized as a key determinant in the development and severity of various age-related degenerative diseases [14], [30].

## Materials and Methods

### Participants

The project protocol and objectives of the study were carefully explained to the research population in their own language by a local translator. As most of the population is illiterate, witness observed oral informed consent was obtained from all participants. In addition, for 615 participants who gave blood for cytokine measurements, an informed consent was obtained either by signature or thumbprint on consent form. This procedure and the whole study were approved by the Medical Ethical Committee of the Ghana Health Service in Ghana, as well as by the Medical Ethical Committee of the Leiden University Medical Center in the Netherlands. In addition, we made use of the data in the International HapMap project (www.hapmap.org) to which a total of 270 people contributed. The Yoruba people of Ibadan, Nigeria data comprises of 30 sets of samples from two parents and an adult child (trio). For Japan and China, 45 unrelated individuals from the Tokyo area and from Beijing, respectively, provided samples. The Centre d'Etude du Polymorphisme Humain (CEPH) data was attained from thirty U.S. trios, which were collected in 1980 from U.S. residents with northern and western European ancestry.

### Study Site

We conducted our study in Garu-Tempane district, a densely populated agricultural area in southeast of the Upper-East region of Ghana, which is inhabited by several tribes, mostly Bimoba (67%) and Kusasi (27%) [15]. The whole Ghana Upper-East region and especially the Garu-Tempane district is underdeveloped with an estimated gross domestic product per head of less than $100 [31]. The research area is situated close to the village of Garu and measures approximately 375 km^2^. People in the research area live in polygamous extended families. The vast majority of the people are farmers and the total agricultural process is done by hand labour. The area is highly endemic for malaria (85% of whole population is infected with *Plasmodium falciparum*), typhoid fever, meningococcal disease and intestinal helminth infections. Hospitals and medical services are only marginally available in the area. Vaccination of children was introduced in the early 1990s, but coverage amongst children is highly variable. It is estimated that about 50% of the children under the age of ten years have been vaccinated at least once against either measles, poliomyelitis, or diphteria-tetanus-pertussis [15]. In 2001, we mapped the research area using a GPS system [15], [31]. Since 2002, we have revisited the area annually to assess population changes. Use of drinking source was determined in 2007.

### Cytokine Production

Cytokine production capacity was assessed in 615 participants by stimulating *ex vivo* whole blood samples with a combination of 10 ng/ml *E. coli* LPS (Sigma-Aldrich, Zwijndrecht, The Netherlands) and 100 µg/ml *S. cerevisea* zymosan (Sigma-Aldrich, Schnelldorf, Germany) [32]. After 24 hours of incubation supernatants were collected and kept at −20°C in Ghana until transported on dry ice to the Netherlands. In the Netherlands all samples were stored at −80°C until cytokine levels were determined by ELISA. Cytokine ELISA for human TNF-α and IL-10 were performed according to manufacturers' guidelines (Central Laboratory of the Blood Transfusion Service, Amsterdam, the Netherlands), with detection limits of 4.0 pg/ml and 3.0 pg/ml respectively.

### DNA Collection and Isolation

For DNA isolation buccal swabs were collected, which were placed in a 15 ml Falcon tube, containing 2.5 ml STE buffer (100 mM NaCl, 10 mM Tris and 10 mM EDTA) with proteinase K (0.05 mg/ml), pronase (0.1 mg/ml) and sodium dodecylsulphate (0.5%), and transported to the Netherlands for DNA isolation. DNA isolation was carried out by a commercial company (BaseClear, Leiden, The Netherlands).

### SNP Selection and Genotyping

We selected 20 SNPs from the *IL10* gene region covering 23 250 bp (chr1:203,320,846-203,344,096) from the HapMap database release #21 (www.hapmap.org) using the Yoruba in Ibadan, Nigeria (Yoruba) data. The Haploview's program Tagger [33] was used to derive a set of tag SNPs from the whole gene region such that each common SNP (≥5%) in that set was captured with r2≥0.8. Besides the SNPs obtained through this approach, polymorphisms that have been associated with a phenotype were included to the analyses. All SNPs were genotyped using mass spectrometry (Sequenom Inc, San Diego, CA, USA), according to the manufacturer's instructions.

### DNA Sequencing

*IL10* gene region was sequenced using 32 PCR fragments that covered in addition to all exons and introns, also upstream and downstream flanking regions, in total 23 250 bp. The data on the sequenced region has been deposited in GenBank (GenBank accession no. GQ405199). The sequencing reactions were performed using Applied Biosystems BigDye (version 3.1) chemistry, and the sequences were resolved using an ABI 3730xl DNA Analyzer. The sequence assembly was performed using the ChromasPro 1.34 software (http://www.technelysium.com.au/ChromasPro.html).

### Statistical Analysis

The program Haploview [33] was used to estimate SNP frequencies and test for Hardy-Weinberg equilibrium. Haplotypes and haplotype frequencies were calculated using PHASE [34]. In all haplotype analyses the posterior probabilities of pairs of haplotypes per participant, as estimated by PHASE, were used as weights. All cytokine levels were log-transformed, since they were not normally distributed, and converted into z-scores ((individual level – mean level)/SD), which were used in further analyses. Association between cytokine production and SNPs or haplotypes was tested with linear regression. The differences in SNP and haplotype frequencies between young, middle-aged and older study participants were tested using linear regression. Cox propositional hazard model was used to calculate mortality risks. All analyses were adjusted for age, sex, socioeconomic status and tribe. Analyses were performed with SPSS version 14.0 (SPSS Inc., Chicago, IL, USA) and STATA version 9 (StataCorp LP, TX, USA) statistical software.

## Supporting Information

Table S1 Location and minor allele frequencies of the *IL10* gene SNPs in the Ghana (n = 4336), Yoruba (n = 90), CEPH (n = 90) and Asian (n = 90) population
(0.07 MB DOC)

Table S2 Association between *IL10* SNPs and production capacity of IL-10 and TNF-α upon co-stimulation with LPS and zymosan (n = 615)
(0.05 MB DOC)

Table S3 *IL10* allele frequency changes over different age-categories and over all ages
(0.07 MB DOC)

Table S4 Minor allele frequencies of *IL10* SNPs for people drinking from wells/rivers (n = 802) or boreholes (n = 3284)
(0.06 MB DOC)

Table S5 Mortality risks for carriers of *IL10* gene SNPs compared to non-carriers for people drinking from wells/rivers (n = 802) or boreholes (n = 3284)
(0.06 MB DOC)

Table S6 Association between *IL10* gene haplotypes and production capacity of IL-10 and TNF-α in a whole blood assay upon co-stimulation with LPS and zymosan (n = 615)
(0.03 MB DOC)

Table S7 *IL10* allele frequency changes over different age-categories and over all ages
(0.03 MB DOC)

Table S8 *IL10* gene haplotype frequencies for people drinking from wells/rivers (n = 802) and boreholes (n = 3284)
(0.03 MB DOC)

Table S9 Mortality risks for carriers of *IL10* gene haplotypes compared to non-carriers for people drinking from wells/rivers (n = 802) and boreholes (n = 3284)
(0.03 MB DOC)

Table S10 *IL10* gene haplotype frequencies for people drinking for their entire lives from wells/rivers (n = 347) or from boreholes (n = 1296)
(0.03 MB DOC)

Table S11 Mortality risks for carriers of *IL10* gene haplotypes compared to non-carriers for people drinking for their entire lives from wells/rivers (n = 347) or from boreholes (n = 1296)
(0.03 MB DOC)

Table S12 Genetic variants identified by re-sequencing the *IL10* gene in 37 individuals
(0.13 MB DOC)

## References

[1] Cooke GS, Hill AV (2001). Genetics of susceptibility to human infectious disease.. Nat Rev Genet.

[2] Crimmins EM, Finch CE (2006). Infection, inflammation, height, and longevity.. Proc Natl Acad Sci U S A.

[3] Le Souef PN, Goldblatt J, Lynch NR (2000). Evolutionary adaptation of inflammatory immune responses in human beings.. Lancet.

[4] van Bodegom D, May L, Meij HJ, Westendorp RG (2007). Regulation of human life histories: the role of the inflammatory host response.. Ann N Y Acad Sci.

[5] Crawley E, Kay R, Sillibourne J, Patel P, Hutchinson I (1999). Polymorphic haplotypes of the interleukin-10 5′ flanking region determine variable interleukin-10 transcription and are associated with particular phenotypes of juvenile rheumatoid arthritis.. Arthritis Rheum.

[6] Franke A, Balschun T, Karlsen TH, Sventoraityte J, Nikolaus S (2008). Sequence variants in IL10, ARPC2 and multiple other loci contribute to ulcerative colitis susceptibility.. Nat Genet.

[7] Smith AJ, Humphries SE (2008). Cytokine and cytokine receptor gene polymorphisms and their functionality.. Cytokine Growth Factor Rev.

[8] van der Pol WL, Huizinga TW, Vidarsson G, van der Linden MW, Jansen MD (2001). Relevance of Fcgamma receptor and interleukin-10 polymorphisms for meningococcal disease.. J Infect Dis.

[9] Westendorp RG, Langermans JA, Huizinga TW, Elouali AH, Verweij CL (1997). Genetic influence on cytokine production and fatal meningococcal disease.. Lancet.

[10] Heiskanen M, Kahonen M, Hurme M, Lehtimaki T, Mononen N (2009). Polymorphism in the IL10 promoter region and early markers of atherosclerosis: The Cardiovascular Risk in Young Finns Study.. Atherosclerosis.

[11] Trompet S, Pons D, de Craen AJ, Slagboom P, Shepherd J (2007). Genetic variation in the interleukin-10 gene promoter and risk of coronary and cerebrovascular events: the PROSPER study.. Ann N Y Acad Sci.

[12] Drenos F, Westendorp RG, Kirkwood TB (2006). Trade-off mediated effects on the genetics of human survival caused by increasingly benign living conditions.. Biogerontology.

[13] Murray CJ, Lopez AD (1997). Mortality by cause for eight regions of the world: Global Burden of Disease Study.. Lancet.

[14] Strong K, Mathers C, Leeder S, Beaglehole R (2005). Preventing chronic diseases: how many lives can we save?. Lancet.

[15] Meij JJ, de Craen AJ, Agana J, Plug D, Westendorp RG (2009). Low-cost interventions accelerate epidemiological transition in Upper East Ghana.. Trans R Soc Trop Med Hyg.

[16] Hu X, Chen J, Wang L, Ivashkiv LB (2007). Crosstalk among Jak-STAT, Toll-like receptor, and ITAM-dependent pathways in macrophage activation.. J Leukoc Biol.

[17] Fiorentino DF, Zlotnik A, Mosmann TR, Howard M, O'Garra A (1991). IL-10 inhibits cytokine production by activated macrophages.. J Immunol.

[18] Shier RP, Dollimore N, Ross DA, Binka FN, Quigley M (1996). Drinking water sources, mortality and diarrhoea morbidity among young children in northern Ghana.. Trop Med Int Health.

[19] Grau GE, Taylor TE, Molyneux ME, Wirima JJ, Vassalli P (1989). Tumor necrosis factor and disease severity in children with falciparum malaria.. N Engl J Med.

[20] Kwiatkowski D, Hill AV, Sambou I, Twumasi P, Castracane J (1990). TNF concentration in fatal cerebral, non-fatal cerebral, and uncomplicated Plasmodium falciparum malaria.. Lancet.

[21] Molyneux ME, Engelmann H, Taylor TE, Wirima JJ, Aderka D (1993). Circulating plasma receptors for tumour necrosis factor in Malawian children with severe falciparum malaria.. Cytokine.

[22] Hartgers FC, Obeng BB, Kruize YC, Duijvestein M, de Breij A (2008). Lower Expression of TLR2 and SOCS-3 Is Associated with Schistosoma haematobium Infection and with Lower Risk for Allergic Reactivity in Children Living in a Rural Area in Ghana.. PLoS Negl Trop Dis.

[23] Maizels RM, Balic A, Gomez-Escobar N, Nair M, Taylor MD (2004). Helminth parasites–masters of regulation.. Immunol Rev.

[24] Jackson JA, Friberg IM, Little S, Bradley JE (2009). Review series on helminths, immune modulation and the hygiene hypothesis: immunity against helminths and immunological phenomena in modern human populations: coevolutionary legacies?. Immunology.

[25] Li JC, Lau AS (2007). A role for mitogen-activated protein kinase and Ets-1 in the induction of interleukin-10 transcription by human immunodeficiency virus-1 Tat.. Immunology.

[26] Shoemaker J, Saraiva M, O'Garra A (2006). GATA-3 directly remodels the IL-10 locus independently of IL-4 in CD4+ T cells.. J Immunol.

[27] Mosser DM, Zhang X (2008). Interleukin-10: new perspectives on an old cytokine.. Immunol Rev.

[28] Ates O, Musellim B, Ongen G, Topal-Sarikaya A (2008). Association between ‘interleukin’ 10 gene (IL10) polymorphisms and systemic sclerosis with interstitial lung involvement.. Rheumatol Int.

[29] Bis JC, Heckbert SR, Smith NL, Reiner AP, Rice K (2008). Variation in inflammation-related genes and risk of incident nonfatal myocardial infarction or ischemic stroke.. Atherosclerosis.

[30] Walker RW, McLarty DG, Kitange HM, Whiting D, Masuki G (2000). Stroke mortality in urban and rural Tanzania. Adult Morbidity and Mortality Project.. Lancet.

[31] van Bodegom D, May L, Kuningas M, Kaptijn R, Thomese F (2009). Socio-economic status by rapid appraisal is highly correlated with mortality risks in rural Africa.. Trans R Soc Trop Med Hyg.

[32] May L, van BD, Kuningas M, Meij JJ, de Craen AJ (2009). Performance of the whole-blood stimulation assay for assessing innate immune activation under field conditions.. Cytokine.

[33] Barrett JC, Fry B, Maller J, Daly MJ (2005). Haploview: analysis and visualization of LD and haplotype maps.. Bioinformatics.

[34] Stephens M, Smith NJ, Donnelly P (2001). A new statistical method for haplotype reconstruction from population data.. Am J Hum Genet.

