# Supplementary material for: Selection for Genetic Variation Inducing Pro-Inflammatory Responses under Adverse Environmental Conditions in a Ghanaian Population
Source: PLoS One. 2009 Nov 11;4(11):e7795. doi: 10.1371/journal.pone.0007795 (PMC2771352; doi:10.1371/journal.pone.0007795)
Supplement: Table S1 — Location and minor allele frequencies of the IL10 gene SNPs in the Ghana (n = 4336), Yoruba (n = 90), CEPH (n = 90) and Asian (n = 90) population (0.07 MB DOC) [file pone.0007795.s001.doc]

**Table S1.** Location and minor allele frequencies of the *IL10* gene SNPs in the Ghana (n=4336), Yoruba (n=90), CEPH (n=90) and Asian (n=90) population

|  | **Alleles^1^** | **Location** | **Minor allele frequency** | | | | **HWE** |
| --- | --- | --- | --- | --- | --- | --- | --- |
| *IL10* SNPs |  |  | Ghana | Yoruba^2^ | CEPH^2^ | Asian^2^ | Ghana |
| rs4072226 | C/T | promoter | 0.456 | 0.458 | 0.456 | 0.017 | 0.865 |
| rs6667202 | C/A | promoter | 0.484 | 0.458 | 0.558 | 0.983 | 0.602 |
| rs6676671 | T/A | promoter | 0.200 | n.a. | n.a. | n.a. | 0.832 |
| rs10494879 | C/G | promoter | 0.284 | 0.283 | 0.458 | 0.056 | **0.025** |
| rs1800890 | T/A | promoter | 0.201 | 0.202 | 0.382 | 0.013 | 0.865 |
| rs6703630 | C/T | promoter | 0.220 | n.a. | n.a. | n.a. | 0.066 |
| rs1800893 | G/A | promoter | 0.284 | 0.347 | 0.534 | 0.056 | 0.196 |
| rs1800896 | A/G | promoter | 0.284 | 0.308 | 0.533 | 0.056 | 0.401 |
| rs1800871 | C/T | promoter | 0.470 | 0.464 | 0.173 | 0.715 | 0.302 |
| rs1800872 | C/A | promoter | 0.472 | 0.483 | 0.202 | 0.716 | **0.034** |
| rs3024490 | G/T | intron | 0.484 | 0.475 | 0.217 | 0.705 | **0.013** |
| rs1554286 | C/T | e/i boundary | 0.468 | 0.466 | 0.169 | 0.699 | 0.157 |
| rs1878672 | C/G | intron | 0.244 | 0.271 | 0.525 | 0.056 | 0.612 |
| rs3024496 | T/C | exon | 0.425 | 0.407 | 0.543 | 0.011 | **0.043** |
| rs3024498 | A/G | exon | 0.083 | 0.108 | 0.288 | 0.000 | 0.129 |
| rs4844553 | C/T | 3’ UTR | 0.096 | 0.092 | 0.058 | 0.000 | 0.084 |
| rs7548373 | G/T | 3’ UTR | 0.297 | 0.250 | 0.067 | 0.000 | 0.190 |
| rs7512090 | C/T | 3’ UTR | 0.132 | 0.125 | 0.067 | 0.000 | 0.084 |
| rs13376708 | G/A | 3’ UTR | 0.327 | 0.330 | 0.123 | 0.050 | 0.273 |
| rs4390174 | A/G | 3’ UTR | 0.282 | 0.286 | 0.317 | 0.209 | 0.630 |

^1^ Major/minor allele; ^2^ Minor allele frequencies from the HapMap database (release nr 23a). n.a. – not available
