# Supplementary material for: Selection for Genetic Variation Inducing Pro-Inflammatory Responses under Adverse Environmental Conditions in a Ghanaian Population
Source: PLoS One. 2009 Nov 11;4(11):e7795. doi: 10.1371/journal.pone.0007795 (PMC2771352; doi:10.1371/journal.pone.0007795)
Supplement: Table S2 — Association between IL10 SNPs and production capacity of IL-10 and TNF-α upon co-stimulation with LPS and zymosan (n = 615) (0.05 MB DOC) [file pone.0007795.s002.doc]

**Table S2.** Association between *IL10* SNPs and production capacity of IL-10 and TNF-α upon co-stimulation with LPS and zymosan (n=615)

|  | **IL-10** | | **TNF-α** | |
| --- | --- | --- | --- | --- |
| *IL10* SNPs | Estimate (s.e.m) | p-value | Estimate (s.e.m) | p-value |
| rs4072226 | -0.08 (0.06) | 0.203 | -0.02 (0.06) | 0.732 |
| rs6667202 | 0.06 (0.06) | 0.305 | 0.00 (0.06) | 0.972 |
| rs6676671 | 0.25 (0.07) | **2.75x10^-04^** | -0.03 (0.07) | 0.705 |
| rs10494879 | 0.18 (0.07) | **5.44x10^-03^** | -0.07 (0.07) | 0.275 |
| rs1800890 | 0.27 (0.07) | **1.03x10^-04^** | -0.06 (0.07) | 0.424 |
| rs6703630 | 0.15 (0.07) | **0.032** | -0.16 (0.07) | **0.029** |
| rs1800893 | 0.32 (0.07) | **3.96x10^-06^** | -0.08 (0.07) | 0.242 |
| rs1800896 | 0.31 (0.06) | **9.98x10^-07^** | -0.04 (0.07) | 0.556 |
| rs1800871 | -0.13 (0.06) | **0.025** | 0.15 (0.06) | **0.013** |
| rs1800872 | -0.14 (0.06) | **0.014** | 0.13 (0.06) | **0.024** |
| rs3024490 | -0.12 (0.06) | **0.029** | 0.16 (0.06) | **3.86x10^-03^** |
| rs1554286 | -0.10 (0.06) | 0.081 | 0.18 (0.06) | **1.86x10^-03^** |
| rs1878672 | 0.34 (0.07) | **5.10x10^-07^** | -0.06 (0.07) | 0.389 |
| rs3024496 | 0.18 (0.06) | **1.64x10^-03^** | -0.12 (0.06) | **0.040** |
| rs3024498 | 0.37 (0.10) | **4.55x10^-04^** | -0.04 (0.11) | 0.693 |
| rs4844553 | 0.15 (0.10) | 0.144 | -0.04 (0.11) | 0.745 |
| rs7548373 | 0.01 (0.07) | 0.916 | -0.09 (0.07) | 0.205 |
| rs7512090 | 0.03 (0.09) | 0.698 | -0.08 (0.09) | 0.369 |
| rs13376708 | -0.08 (0.06) | 0.195 | -0.09 (0.06) | 0.138 |
| rs4390174 | -0.06 (0.06) | 0.358 | 0.06 (0.06) | 0.316 |

Linear regression adjusted for age, sex, tribe, and socio-economic status. Data presented as z-scores with standard errors (s.e.m)
