# Supplementary material for: Selection for Genetic Variation Inducing Pro-Inflammatory Responses under Adverse Environmental Conditions in a Ghanaian Population
Source: PLoS One. 2009 Nov 11;4(11):e7795. doi: 10.1371/journal.pone.0007795 (PMC2771352; doi:10.1371/journal.pone.0007795)
Supplement: Table S3 — IL10 allele frequency changes over different age-categories and over all ages (0.07 MB DOC) [file pone.0007795.s003.doc]

**Table S3.** *IL10* allele frequency changes over different age-categories and over all ages

|  | **Minor allele frequency** | | | |  | **Change with age** | |
| --- | --- | --- | --- | --- | --- | --- | --- |
| *IL10* SNPs | ≤5 years (n=1014) | 20-45 years, (n=1462) | ≥60 years (n=727) | p-value |  | Difference (s.e.m)  (n=4336) | p-value |
| rs4072226 | 0.453 | 0.456 | 0.443 | 0.560 |  | -0.26 (0.58) | 0.650 |
| rs6667202 | 0.490 | 0.475 | 0.511 | 0.294 |  | 0.66 (0.59) | 0.258 |
| rs6676671 | 0.219 | 0.191 | 0.210 | 0.426 |  | -0.53 (0.71) | 0.457 |
| rs10494879 | 0.312 | 0.282 | 0.288 | 0.130 |  | -0.79 (0.65) | 0.228 |
| rs1800890 | 0.227 | 0.194 | 0.203 | 0.066 |  | -1.10 (0.71) | 0.123 |
| rs6703630 | 0.229 | 0.222 | 0.212 | 0.287 |  | -0.83 (0.70) | 0.238 |
| rs1800893 | 0.291 | 0.286 | 0.243 | **4.76x10^-3^** |  | -1.77 (0.69) | **0.010** |
| rs1800896 | 0.305 | 0.275 | 0.272 | **0.018** |  | -1.22 (0.65) | 0.059 |
| rs1800871 | 0.441 | 0.469 | 0.490 | **4.73x10^-3^** |  | 1.38 (0.58) | **0.017** |
| rs1800872 | 0.447 | 0.470 | 0.489 | **0.015** |  | 1.17 (0.58) | **0.045** |
| rs3024490 | 0.457 | 0.482 | 0.501 | **0.014** |  | 1.07 (0.56) | 0.057 |
| rs1554286 | 0.433 | 0.471 | 0.490 | **1.38x10^-4^** |  | 1.52 (0.57) | **0.008** |
| rs1878672 | 0.254 | 0.233 | 0.232 | 0.110 |  | -0.90 (0.67) | 0.179 |
| rs3024496 | 0.451 | 0.416 | 0.419 | 0.060 |  | -0.86 (0.57) | 0.135 |
| rs3024498 | 0.087 | 0.083 | 0.078 | 0.422 |  | -1.29 (1.03) | 0.214 |
| rs4844553 | 0.099 | 0.090 | 0.079 | 0.056 |  | -1.56 ()0.96 | 0.105 |
| rs7548373 | 0.309 | 0.293 | 0.292 | 0.376 |  | -0.55 (0.65) | 0.393 |
| rs7512090 | 0.127 | 0.134 | 0.117 | 0.426 |  | -0.72 (0.84) | 0.393 |
| rs13376708 | 0.325 | 0.327 | 0.333 | 0.716 |  | 0.48 (0.62) | 0.437 |
| rs4390174 | 0.272 | 0.285 | 0.293 | 0.209 |  | 0.84 (0.64) | 0.187 |

p-value calculated using linear regression adjusted for sex, socioeconomic status and tribe
