# Supplementary material for: Selection for Genetic Variation Inducing Pro-Inflammatory Responses under Adverse Environmental Conditions in a Ghanaian Population
Source: PLoS One. 2009 Nov 11;4(11):e7795. doi: 10.1371/journal.pone.0007795 (PMC2771352; doi:10.1371/journal.pone.0007795)
Supplement: Table S4 — Minor allele frequencies of IL10 SNPs for people drinking from wells/rivers (n = 802) or boreholes (n = 3284) (0.06 MB DOC) [file pone.0007795.s004.doc]

**Table S4.** Minor allele frequencies of *IL10* SNPs for people drinking from wells/rivers (n=802) or boreholes (n=3284)

|  | **Minor allele frequency** | |  |
| --- | --- | --- | --- |
| *IL10* SNPs | Wells/rivers | Boreholes | p-value |
| rs4072226 | 0.476 | 0.450 | 0.058 |
| rs6667202 | 0.468 | 0.490 | 0.120 |
| rs6676671 | 0.198 | 0.201 | 0.768 |
| rs10494879 | 0.275 | 0.290 | 0.218 |
| rs1800890 | 0.196 | 0.204 | 0.476 |
| rs6703630 | 0.209 | 0.223 | 0.222 |
| rs1800893 | 0.273 | 0.283 | 0.399 |
| rs1800896 | 0.287 | 0.283 | 0.771 |
| rs1800871 | 0.492 | 0.462 | **0.028** |
| rs1800872 | 0.494 | 0.463 | **0.022** |
| rs3024490 | 0.505 | 0.475 | **0.036** |
| rs1554286 | 0.492 | 0.461 | **0.027** |
| rs1878672 | 0.249 | 0.241 | 0.481 |
| rs3024496 | 0.419 | 0.428 | 0.522 |
| rs3024498 | 0.087 | 0.083 | 0.636 |
| rs4844553 | 0.100 | 0.093 | 0.377 |
| rs7548373 | 0.288 | 0.298 | 0.438 |
| rs7512090 | 0.128 | 0.131 | 0.752 |
| rs13376708 | 0.300 | 0.332 | **0.014** |
| rs4390174 | 0.286 | 0.281 | 0.716 |

p-value calculated using Linear-by-Linear Association test with df=1
