# Supplementary material for: Selection for Genetic Variation Inducing Pro-Inflammatory Responses under Adverse Environmental Conditions in a Ghanaian Population
Source: PLoS One. 2009 Nov 11;4(11):e7795. doi: 10.1371/journal.pone.0007795 (PMC2771352; doi:10.1371/journal.pone.0007795)
Supplement: Table S5 — Mortality risks for carriers of IL10 gene SNPs compared to non-carriers for people drinking from wells/rivers (n = 802) or boreholes (n = 3284) (0.06 MB DOC) [file pone.0007795.s005.doc]

**Table S5.** Mortality risks for carriers of *IL10* gene SNPs compared to non-carriers for people drinking from wells/rivers (n=802) or boreholes (n=3284)

|  | **Wells/rivers** | |  | **Boreholes** | |  | **Interaction** |
| --- | --- | --- | --- | --- | --- | --- | --- |
| *IL10* SNPs | HR (95% CI) | p-value |  | HR (95% CI) | p-value |  | p_interaction_ |
| rs4072226 | 0.79 (0.43-1.43) | 0.431 |  | 0.98 (0.72-1.34) | 0.905 |  | 0.613 |
| rs6667202 | 0.79 (0.43-1.46) | 0.454 |  | 1.47 (1.00-2.17) | **0.049** |  | 0.099 |
| rs6676671 | 1.64 (0.97-2.77) | 0.063 |  | 0.83 (0.61-1.12) | 0.226 |  | **0.029** |
| rs10494879 | 1.22 (0.72-2.05) | 0.466 |  | 0.92 (0.69-1.23) | 0.580 |  | 0.361 |
| rs1800890 | 1.61 (0.96-2.71) | 0.073 |  | 0.79 (0.58-1.08) | 0.143 |  | **0.023** |
| rs6703630 | 1.36 (0.78-2.35) | 0.276 |  | 0.99 (0.75-1.32) | 0.967 |  | 0.264 |
| rs1800893 | 1.30 (0.75-2.26) | 0.351 |  | 0.91 (0.68-1.22) | 0.516 |  | 0.270 |
| rs1800896 | 1.51 (0.87-2.60) | 0.141 |  | 0.88 (0.65-1.18) | 0.390 |  | 0.099 |
| rs1800871 | 0.72 (0.38-1.36) | 0.313 |  | 1.44 (1.01-2.04) | **0.042** |  | 0.054 |
| rs1800872 | 0.89 (0.46-1.71) | 0.725 |  | 1.47 (1.03-2.10) | **0.036** |  | 0.149 |
| rs3024490 | 0.74 (0.39-1.38) | 0.338 |  | 1.51 (1.06-2.14) | **0.023** |  | **0.040** |
| rs1554286 | 0.83 (0.44-1.55) | 0.557 |  | 1.49 (1.05-2.11) | **0.027** |  | 0.105 |
| rs1878672 | 1.61 (0.93-2.79) | 0.087 |  | 0.81 (0.60-1.08) | 0.155 |  | **0.031** |
| rs3024496 | 1.33 (0.69-2.55) | 0.390 |  | 1.08 (0.78-1.48) | 0.652 |  | 0.544 |
| rs3024498 | 1.20 (0.57-2.52) | 0.635 |  | 0.91 (0.60-1.37) | 0.643 |  | 0.509 |
| rs4844553 | 1.48 (0.82-2.67) | 0.195 |  | 0.68 ()0.44-1.06 | 0.092 |  | **0.040** |
| rs7548373 | 0.94 (0.53-1.68) | 0.830 |  | 1.00 (0.74-1.36) | 0.990 |  | 0.944 |
| rs7512090 | 1.64 (0.90-2.98) | 0.106 |  | 0.75 (0.52-1.09) | 0.136 |  | **0.027** |
| rs13376708 | 1.12 (0.62-2.01) | 0.713 |  | 0.80 (0.60-1.05) | 0.111 |  | 0.308 |
| rs4390174 | 1.47 (0.83-2.58) | 0.183 |  | 1.09 (0.81-1.46) | 0.582 |  | 0.443 |

Data presented and hazard ratios (HR) with 95 % confidence intervals (CI). Cox proportional hazard model adjusted for age, sex, tribe and socio-economic status
