# Supplementary material for: Selection for Genetic Variation Inducing Pro-Inflammatory Responses under Adverse Environmental Conditions in a Ghanaian Population
Source: PLoS One. 2009 Nov 11;4(11):e7795. doi: 10.1371/journal.pone.0007795 (PMC2771352; doi:10.1371/journal.pone.0007795)
Supplement: Table S6 — Association between IL10 gene haplotypes and production capacity of IL-10 and TNF-α in a whole blood assay upon co-stimulation with LPS and zymosan (n = 615) (0.03 MB DOC) [file pone.0007795.s006.doc]

**Table S6.** Association between *IL10* gene haplotypes and production capacity of IL-10 and TNF-α in a whole blood assay upon co-stimulation with LPS and zymosan (n=615)

|  | **IL-10** | |  | **TNF-α** | |
| --- | --- | --- | --- | --- | --- |
| *IL10* haplotypes | Estimate (s.e.m) | p-value |  | Estimate (s.e.m) | p-value |
| Haplotype 1 | -0.11 (0.05) | **0.028** |  | 0.18 (0.06) | **1.39x10^-3^** |
| Haplotype 2 | 0.17 (0.10) | 0.081 |  | -0.01 (0.12) | 0.898 |
| Haplotype 3 | 0.38 (0.10) | **1.67x10^-4^** |  | 0.02 (0.10) | 0.807 |
| Haplotype 4 | -0.07 (0.10) | 0.523 |  | -0.11 (0.12) | 0.382 |
| Haplotype 5 | -0.15 (0.12) | 0.206 |  | -0.21 (0.13) | 0.107 |

Linear regression adjusted for age, sex, tribe, and socio-economic status. Data presented as z-scores with standard errors (s.e.m)
