# Supplementary material for: Selection for Genetic Variation Inducing Pro-Inflammatory Responses under Adverse Environmental Conditions in a Ghanaian Population
Source: PLoS One. 2009 Nov 11;4(11):e7795. doi: 10.1371/journal.pone.0007795 (PMC2771352; doi:10.1371/journal.pone.0007795)
Supplement: Table S7 — IL10 allele frequency changes over different age-categories and over all ages (0.03 MB DOC) [file pone.0007795.s007.doc]

**Table S7.** *IL10* allele frequency changes over different age-categories and over all ages

|  | **Haplotype frequency** | | | |  | **Change with age** | |
| --- | --- | --- | --- | --- | --- | --- | --- |
| *IL10* haplotypes | ≤5 years  (n=1014) | 20-45 years  (n=1462) | ≥ 60 years  (n=727) | p-value |  | Difference (s.e.m)  (n=4336) | p-value |
| Haplotype 1 | 0.399 | 0.449 | 0.455 | **2.46x10^-6^** |  | 3.26 (0.82) | **6.87x10^-5^** |
| Haplotype 2 | 0.085 | 0.082 | 0.078 | 0.413 |  | -0.35 (1.05) | 0.739 |
| Haplotype 3 | 0.080 | 0.080 | 0.068 | 0.944 |  | -0.15 (1.05) | 0.889 |
| Haplotype 4 | 0.067 | 0.070 | 0.073 | 0.122 |  | 1.80 (1.13) | 0.112 |
| Haplotype 5 | 0.053 | 0.061 | 0.046 | 0.988 |  | -0.19 (1.23) | 0.879 |

p-value calculated using linear regression adjusted for sex, socioeconomic status and tribe
