# Supplementary material for: Selection for Genetic Variation Inducing Pro-Inflammatory Responses under Adverse Environmental Conditions in a Ghanaian Population
Source: PLoS One. 2009 Nov 11;4(11):e7795. doi: 10.1371/journal.pone.0007795 (PMC2771352; doi:10.1371/journal.pone.0007795)
Supplement: Table S8 — IL10 gene haplotype frequencies for people drinking from wells/rivers (n = 802) and boreholes (n = 3284) (0.03 MB DOC) [file pone.0007795.s008.doc]

**Table S8.** *IL10* gene haplotype frequencies for people drinking from wells/rivers (n=802) and boreholes (n=3284)

|  | **Haplotype frequency** | |  |
| --- | --- | --- | --- |
| *IL10* haplotypes | Wells/rivers | Boreholes | p-value |
| Haplotype 1 | 0.467 | 0.434 | **8.20x10^-3^** |
| Haplotype 2 | 0.085 | 0.079 | 0.659 |
| Haplotype 3 | 0.087 | 0.081 | 0.466 |
| Haplotype 4 | 0.066 | 0.071 | 0.464 |
| Haplotype 5 | 0.045 | 0.057 | 0.056 |

p-value calculated using logistic regression adjusted for age, sex, socioeconomic status and tribe
