# Supplementary material for: Selection for Genetic Variation Inducing Pro-Inflammatory Responses under Adverse Environmental Conditions in a Ghanaian Population
Source: PLoS One. 2009 Nov 11;4(11):e7795. doi: 10.1371/journal.pone.0007795 (PMC2771352; doi:10.1371/journal.pone.0007795)
Supplement: Table S9 — Mortality risks for carriers of IL10 gene haplotypes compared to non-carriers for people drinking from wells/rivers (n = 802) and boreholes (n = 3284) (0.03 MB DOC) [file pone.0007795.s009.doc]

**Table S9.** Mortality risks for carriers of *IL10* gene haplotypes compared to non-carriers for people drinking from wells/rivers (n=802) and boreholes (n=3284)

|  | **Wells/rivers** | |  | **Boreholes** | |  | **Interaction** |
| --- | --- | --- | --- | --- | --- | --- | --- |
| *IL10* haplotypes | HR (95% CI) | p-value |  | HR (95% CI) | p-value |  | p_interaction_ |
| Haplotype 1 | 0.67 (0.38-1.18) | 0.170 |  | 1.47 (1.08-1.99) | **0.013** |  | **0.013** |
| Haplotype 2 | 1.51 (0.83-2.78) | 0.180 |  | 0.67 (0.43-1.05) | 0.080 |  | **0.037** |
| Haplotype 3 | 1.35 (0.70-2.60) | 0.367 |  | 1.06 (0.73-1.54) | 0.757 |  | 0.518 |
| Haplotype 4 | 0.53 (0.21-1.32) | 0.171 |  | 1.51 (1.04-2.18) | **0.028** |  | **0.039** |
| Haplotype 5 | 2.10 (1.10-4.01) | **0.025** |  | 0.77 (0.46-1.29) | 0.320 |  | **0.011** |

Data presented and hazard ratios (HR) with 95 % confidence intervals (CI). Cox proportional hazard model adjusted for age, sex, tribe and socio-economic status
