# Supplementary material for: Selection for Genetic Variation Inducing Pro-Inflammatory Responses under Adverse Environmental Conditions in a Ghanaian Population
Source: PLoS One. 2009 Nov 11;4(11):e7795. doi: 10.1371/journal.pone.0007795 (PMC2771352; doi:10.1371/journal.pone.0007795)
Supplement: Table S10 — IL10 gene haplotype frequencies for people drinking for their entire lives from wells/rivers (n = 347) or from boreholes (n = 1296) (0.03 MB DOC) [file pone.0007795.s010.doc]

**Table S10.** *IL10* gene haplotype frequencies for people drinking for their entire lives from wells/rivers (n=347) or from boreholes (n=1296)

|  | **Haplotype frequency** | |  |
| --- | --- | --- | --- |
| *IL10* haplotypes | Wells/rivers | Boreholes | p-value |
| Haplotype 1 | 0.452 | 0.402 | 0.062 |
| Haplotype 2 | 0.090 | 0.087 | 0.698 |
| Haplotype 3 | 0.079 | 0.087 | 0.966 |
| Haplotype 4 | 0.053 | 0.068 | 0.204 |
| Haplotype 5 | 0.036 | 0.055 | 0.060 |

p-value calculated using logistic regression adjusted for age, sex, socio-economic status and tribe
