# Supplementary material for: Selection for Genetic Variation Inducing Pro-Inflammatory Responses under Adverse Environmental Conditions in a Ghanaian Population
Source: PLoS One. 2009 Nov 11;4(11):e7795. doi: 10.1371/journal.pone.0007795 (PMC2771352; doi:10.1371/journal.pone.0007795)
Supplement: Table S11 — Mortality risks for carriers of IL10 gene haplotypes compared to non-carriers for people drinking for their entire lives from wells/rivers (n = 347) or from boreholes (n = 1296) (0.03 MB DOC) [file pone.0007795.s011.doc]

**Table S11.** Mortality risks for carriers of *IL10* gene haplotypes compared to non-carriers for people drinking for their entire lives from wells/rivers (n=347) or from boreholes (n=1296)

|  | **Wells/rivers** | |  | **Boreholes** | |  | **Interaction** |
| --- | --- | --- | --- | --- | --- | --- | --- |
| *IL10* haplotypes | HR (95% CI) | p-value |  | HR (95% CI) | p-value |  | p_interaction_ |
| Haplotype 1 | 0.97 (0.32-2.89) | 0.951 |  | 1.33 (0.79-2.25) | 0.281 |  | 0.731 |
| Haplotype 2 | 1.31 (0.35-4.87) | 0.685 |  | 0.96 (0.48-1.90) | 0.913 |  | 0.568 |
| Haplotype 3 | 1.83 (0.62-5.44) | 0.277 |  | 1.31 (0.71-2.44) | 0.388 |  | 0.653 |
| Haplotype 4 | - | - |  | 1.41 (0.71-2.81) | 0.328 |  | - |
| Haplotype 5 | 1.51 (0.40-5.65) | 0.539 |  | 0.73 (0.97-1.01) | 0.219 |  | 0.434 |

Data presented and hazard ratios (HR) with 95 % confidence intervals (CI). Cox proportional hazard model adjusted for age, sex, tribe and socio-economic status
