# Supplementary material for: Selection for Genetic Variation Inducing Pro-Inflammatory Responses under Adverse Environmental Conditions in a Ghanaian Population
Source: PLoS One. 2009 Nov 11;4(11):e7795. doi: 10.1371/journal.pone.0007795 (PMC2771352; doi:10.1371/journal.pone.0007795)
Supplement: Table S12 — Genetic variants identified by re-sequencing the IL10 gene in 37 individuals (0.13 MB DOC) [file pone.0007795.s012.doc]

**Table S12.** Genetic variants identified by re-sequencing the *IL10* gene in 37 individuals

| # | **rs-number** | **Alleles** | **Position (bp)** | **Location** | **Sequence** | **MAF** |
| --- | --- | --- | --- | --- | --- | --- |
| 1 | IL10_1 | G/A | 205021474 | promoter | agaggtaaggaccagaatgg[g/a]caggatgatgtccttctgag | 0.081 |
| 2 | rs12123181 | C/T | 205021189 | promoter | gaactactgacctagtgatc[c/t]gcccatctcggcctcccaaa | 0.297 |
| 3 | IL10_2 | G/A | 205021149 | promoter | agtgctgggattacaggcgt[g/a]agccaccgcgcctggcccct | 0.297 |
| 4 | IL10_3 | C/T | 205021094 | promoter | aaggtttctggaaggtgttg[c/t]tcctgtaggggtactgcagt | 0.014 |
| 5 | IL10_4 | G/A | 205020288 | promoter | gagaaccttggatgtgccca[g/a]aaccatgctgagtactgagc | 0.027 |
| 6 | GAGdel | GAGdel | 205020019 | promoter | ctgaaggatgaagacaggag[gag]agcaggaagggagaactaaa | 0.014 |
| 7 | Gains | GAins | 205019825 | promoter | tctctttaaaaaaaaaaaaa[ga]gagagagagagcatgcggat | 0.041 |
| 8 | IL10_5 | T/A | 205020019 | promoter | aagtagcaacagagtcatga[t/a]cagattggtgcttggaaata | 0.095 |
| 9 | rs6676671 | T/A | 205019371 | promoter | aaaatgggtccagtgggcca[t/a]cactggtatggaggctgcaa | 0.189 |
| 10 | IL10_6 | C/T | 205019008 | promoter | gcttggtgttccaaaggcca[c/t]gagcggttttagaccctgga | 0.054 |
| 11 | IL10_7 | C/T | 205018907 | promoter | cttctaccaacagatagtag[c/t]ttgaaccagacagtcattgc | 0.014 |
| 12 | rs10494879 | C/G | 205018827 | promoter | aaatgtttatgaagaaaaat[c/g]tggaggttctggtgatagat | 0.324 |
| 13 | IL10_8 | A/C | 205018343 | promoter | catgcagacagagcagaaac[a/c]actatggtcctctgggtggc | 0.014 |
| 14 | rs17015767 | C/G | 205018021 | promoter | taaacttctggcccaccagg[c/g]gtttattctgaagtacccca | 0.311 |
| 15 | rs12122923 | G/A | 205018020 | promoter | aaacttctggcccaccaggc[g/a]tttattctgaagtaccccag | 0.122 |
| 16 | rs17015763 | G/A | 205017842 | promoter | cctctgtcctaggagctgag[g/a]aagcttattcgagggaatac | 0.068 |
| 17 | rs1800890 | T/A | 205015988 | promoter | acatcccccactggaaaaat[t/a]catttaaaatcagtataata | 0.311 |
| 18 | rs6703630 | C/T | 205015262 | promoter | tctgggaggccaaggcgggc[c/t]gatcataaggtcaggagatc | 0.392 |
| 19 | rs1800891 | A/G | 205015189 | promoter | tctctaataaaaatacaaaa[a/g]aaaataaaaaaacttagccg | 0.108 |
| 20 | rs6693899 | C/A | 205015176 | promoter | tacaaaaaaaaataaaaaaa[c/a]ttagccgggcgtggtgccag | 0.405 |
| 21 | IL10_9 | C/T | 205014629 | promoter | tcggcagggcatggtgtgta[c/t]atgaatgataaccacgtagg | 0.014 |
| 22 | rs1800893 | G/A | 205013790 | promoter | ggcctcctgcacctaggtca[g/a]tgttcctcccagttacagtc | 0.432 |
| 23 | rs1800896 | A/G | 205013520 | promoter | ctactaaggcttctttggga[a/g]ggggaagtagggataggtaa | 0.432 |
| 24 | rs1800871 | C/T | 205013257 | promoter | cccttgtacaggtgatgtaa[c/t]atctctgtgcctcagtttgc | 0.486 |
| 25 | rs1800872 | C/A | 205013030 | promoter | catcctgtgaccccgcctgt[c/a]ctgtaggaagccagtctctg | 0.486 |
| 26 | IL10_10 | C/A | 205013010 | intron1 | ctccatccccaacacctatt[c/a]ccccaaacttaaattcttaa | 0.344 |
| 27 | rs2222202 | C/T | 205012004 | intron1 | ttataaagtaaatgcgttct[c/t]tctcgtgctgagaaacttat | 0.432 |
| 28 | rs3024490 | G/T | 205011934 | intron1 | ggctaggagaagtaaagaaa[g/t]gtctgattcgaggtgaagat | 0.486 |
| 29 | rs3024491 | G/T | 205011669 | intron1 | cataggtgtcccttaaagcc[g/t]aatgtagctccgcagaaaga | 0.432 |
| 30 | rs1518110 | T/G | 205011484 | intron1 | ttttgggccagagccaattt[t/g]atttaaaaaaaaaaatctct | 0.473 |
| 31 | rs1518111 | A/G | 205011268 | intron3 (boundary) | gcatgattaagggaagggag[a/g]ctctgcttcctgattgcagg | 0.473 |
| 32 | rs1554286 | C/T | 205010856 | intron3 (boundary) | gtgtaagtagcagatcagtt[c/t]tttcccttgcagctgccccc | 0.473 |
| 33 | rs1878672 | C/G | 205010336 | intron3 | tctccaactgaaatgatgcc[c/g]tcactactaatggtttcccc | 0.419 |
| 34 | rs3024494 | G/A | 205009974 | intron3 | ctctctgggagctgtgaggc[g/a]aggcatttggataaatctgg | 0.149 |
| 35 | rs3024496 | T/C | 205008487 | 3'UTR | aaaccttattgtacctctct[t/c]atagaatatttattacctct | 0.419 |
| 36 | rs3024497 | A/G | 205008413 | 3'UTR | agcttctctgtgaacgattt[a/g]gaaagaagcccaatattata | 0.014 |
| 37 | rs3024498 | A/G | 205008152 | 3'UTR | cttggggcttcctaactgct[a/g]caaatactcttaggaagaga | 0.311 |
| 38 | IL10_11 | G/A | 205007858 | 3'UTR | taaaaatacaaaaattagcc[g/a]ggcatggtggcgcgcacctg | 0.041 |
| 39 | rs3024502 | G/A | 205006933 | downstream | ggcgtgagccaccacacccg[g/a]ccaggggaagtttttagggt | 0.432 |
| 40 | rs3024503 | A/G | 205006844 | downstream | agggcttctggcactaggag[a/g]tgttcgcagtgcgtatagtt | 0.014 |
| 41 | rs61815632 | T/C | 205005062 | downstream | actgcatcattcttctttcc[t/c]aatataattctgtttgtatc | 0.324 |
| 42 | IL10_12 | A/G | 205004740 | downstream | gtctgggagtgacaactagg[a/g]attccatctcaggatttcta | 0.014 |
| 43 | IL10_13 | C/A | 205004340 | downstream | tattttaaaacaatcacaac[c/a]ttttaagaatctagaagaaa | 0.014 |
| 44 | IL10_14 | A/G | 205004327 | downstream | tcacaaccttttaagaatct[a/g]gaagaaatagttttgcgctc | 0.014 |
| 45 | IL10_15 | C/T | 205003953 | downstream | gagagcttgctgtcttcccc[c/t]cactacccccaagtgtgctc | 0.054 |
| 46 | rs6673928 | C/A | 205003868 | downstream | caagccaagagaagaggact[c/a]agaatgaaacctgccttgcc | 0.311 |
| 47 | Tins | Tins | 205003634 | downstream | gttttgatgggatttttttt[t]caacaagcctttattctcta | 0.054 |
| 48 | IL10_16 | T/G | 205003621 | downstream | tttttttcaacaagccttta[t/g]tctctaagaaaagcatccat | 0.014 |
| 49 | IL10_17 | C/T | 205002329 | downstream | cctggggacaagagtgacag[c/t]gcttatagcaggtgatgggg | 0.014 |
| 50 | IL10_18 | C/T | 205001477 | downstream | tgtgtattatcaacagctcc[c/t]tcccccagcagaattgagct | 0.014 |
| 51 | IL10_19 | A/G | 205001376 | downstream | gacataaaagacctaaggaa[a/g]gtgacacagcgtacctctga | 0.014 |
| 52 | rs4844553 | C/T | 205000986 | downstream | ctccctaccaccatttctgg[c/t]cggaaagagtaaatggtttg | 0.122 |
| 53 | rs7548373 | G/T | 205000113 | downstream | acaagatacacacttattca[g/t]tcagaacttattatgctcca | 0.122 |
| 54 | rs7519318 | A/G | 205000010 | downstream | ggatttcttttaatttttta[a/g]atagagatgggggtctcact | 0.122 |
| 55 | Cdel | Cdel | 204999977 | downstream | gtctcactatgttgcccagg[c]tggatttgaactcctaggtg | 0.014 |
| 56 | rs7512090 | C/T | 204999960 | downstream | aggctggatttgaactccta[c/t]gtgcaagtgatcctccttcc | 0.122 |
| 57 | IL10_20 | G/A | 204999623 | downstream | agaaggggtttatctctgga[g/a]caaaggtaaatttgtaccaa | 0.014 |
| 58 | Ains | Ains | 204999373 | downstream | taggctatggtcaaaaattt[a]gactctaagagcaaagggaa | 0.068 |

bp – base pair; MAF – minor allele frequency
